# Increment of specific heat capacity of solar salt with SiO_2_ nanoparticles

**DOI:** 10.1186/1556-276X-9-582

**Published:** 2014-10-20

**Authors:** Patricia Andreu-Cabedo, Rosa Mondragon, Leonor Hernandez, Raul Martinez-Cuenca, Luis Cabedo, J Enrique Julia

**Affiliations:** 1 Departamento de Ingeniería Mecánica y Construcción, Universitat Jaume I. Campus de Riu Sec, Castellón de la Plana, Spain; 2 Polymers and Advanced Materials Group (PIMA), Universitat Jaume I. Campus de Riu Sec, Castellón de la Plana, Spain

**Keywords:** Nanofluid, Solar salt, Specific heat capacity, Thermal energy storage

## Abstract

Thermal energy storage (TES) is extremely important in concentrated solar power (CSP) plants since it represents the main difference and advantage of CSP plants with respect to other renewable energy sources such as wind, photovoltaic, etc. CSP represents a low-carbon emission renewable source of energy, and TES allows CSP plants to have energy availability and dispatchability using available industrial technologies. Molten salts are used in CSP plants as a TES material because of their high operational temperature and stability of up to 500°C. Their main drawbacks are their relative poor thermal properties and energy storage density. A simple cost-effective way to improve thermal properties of fluids is to dope them with nanoparticles, thus obtaining the so-called salt-based nanofluids. In this work, solar salt used in CSP plants (60% NaNO_3_ + 40% KNO_3_) was doped with silica nanoparticles at different solid mass concentrations (from 0.5% to 2%). Specific heat was measured by means of differential scanning calorimetry (DSC). A maximum increase of 25.03% was found at an optimal concentration of 1 wt.% of nanoparticles. The size distribution of nanoparticle clusters present in the salt at each concentration was evaluated by means of scanning electron microscopy (SEM) and image processing, as well as by means of dynamic light scattering (DLS). The cluster size and the specific surface available depended on the solid content, and a relationship between the specific heat increment and the available particle surface area was obtained. It was proved that the mechanism involved in the specific heat increment is based on a surface phenomenon. Stability of samples was tested for several thermal cycles and thermogravimetric analysis at high temperature was carried out, the samples being stable.

**PACS:**

65.: Thermal properties of condensed matter; 65.20.-w: Thermal properties of liquids; 65.20.Jk: Studies of thermodynamic properties of specific liquids

## Background

The global warming and terawatt challenge are probably two of the most complex issues that humanity have to face during the twenty-first century [1]. In order to comply with these assessments, improvement of energy efficiency and conservation as well as development of low-carbon energy technologies must play a lead role. The development of cost-effective energy storage technologies is one of the key parameters for improving energy management and providing availability and dispatchability to low-carbon renewable energy sources [2]. Energy storage technologies can bridge temporal and geographical gaps between energy demand and supply. While some energy storage technologies are mature, most of them are still in the early stages of development and additional research efforts are needed.

Ninety percent of the current energy budget centers on heat conversion, transmission, and storage. Consequently, thermal energy storage (TES) systems play a key role in energy storage. Affordable TES systems (typically for renewable energy sources, waste heat, or surplus energy production) can replace heat and cold production from fossil fuels, reduce greenhouse gas emissions, and lower the need for costly peak power and heat production capacity [3].

Inorganic salts are used as sensible, latent, and thermochemical materials in TES systems in a wide range of temperatures from -5°C to more than 1,000°C. The main advantages of TES systems based on inorganic salts are their competitive costs, high stability at high temperatures, as well as being environmentally friendly. Their main disadvantages are the low thermal properties and corrosion problems. Actual commercial high-temperature TES systems are based on solar salt (eutectic mixture of NaNO_3_ and KNO_3_) and its main application is in concentrated solar power (CSP) plants in which solar salt is used as a sensible TES in two tank configuration as well as, in some cases, heat transfer fluid.

The concept of adding small solid particles to a base fluid to increase the thermal properties of the suspension has been practiced for a long time [4]. However, most of the studies were performed using suspensions of millimeter- or micrometer-sized particles, which led to problems such as poor suspension stability and channel clogging, which limits its practical applicability. In 1995, Choi proposed the use of nanofluids to increase the thermal conductivity of heat transfer fluids [5]. Nanofluids are defined as dilute suspensions with solid particles smaller than 100 nm. Nanofluids present some important advantages over the conventional colloidal suspensions such as high stability, reduced particle clogging, and high heat transfer capabilities. Up to now, research has been focused on the characterization and modeling of the thermal conductivity, viscosity, heat transfer coefficient, and pressure drop of water-based nanofluids. Increments of up to 30% in thermal conductivity can be found using high nanoparticle loads. The main drawback of water and thermal oil-based nanofluids is their high viscosity (increase of 5 to 10 times). Efforts to reduce nanofluid viscosity and stability at high temperatures are still needed [6].

The effect of adding nanoparticles on the specific heat capacity (Cp) of thermal storage fluids is still under early research. According to the mixture rule (see Equation 1), the addition of nanoparticles presenting a lower value of Cp than the base fluid results in a decrease in the specific heat of the nanofluid [7],

(1) $${Cp}_{nf}=\frac{\phi \left(\rho_{np}{Cp}_{np}\right)+\left(1-\phi \right)\left(\rho_{bf}{Cp}_{bf}\right)}{\phi \left(\rho_{np}\right)+\left(1-\phi \right)\left(\rho_{bf}\right)}$$

where ϕ is the volume fraction of nanoparticles, ρ the density and the subscripts nf, np, and bf refer to the nanofluid, nanoparticle, and base fluid, respectively.

This behavior can be found when water and ethylene glycol are used as the base fluid. In the case of using oil as base fluid, it is possible to find some works with increments in Cp of up to 50% [8]. In addition, recent works have shown that the Cp of salts (organic and inorganic) is increased when nanoparticles are added in an appropriate way. Nanofluids that have salts as their base fluid are called salt-based nanofluids or nanosalts.

The first works dealing with inorganic salt-based nanofluids date from 2010 [9]. Table 1 summarizes the experimental works related with salt-based nanofluids. Most of the published works have been focused in eutectic mixtures of carbonate (Li_2_CO_3_ and K_2_CO_3_) [10-15], chloride (BaCl_2_, NaCl, CaCl_2_, and LiCl) [16], and nitrate (NaNO_3_ and KNO_3_) [17-20]. In most cases, SiO_2_ nanoparticles have been used [10-13,16,18,19]. In addition, Al_2_O_3_[15,17,19,20], TiO_2_[19], and graphite [14] nanoparticles have been tested.

**Table 1:** Summary of the previous published works and present work in salt-based nanofluids

**Authors**	**Year**	**Salt composition**	**Nanoparticles**	**Preparation method**	**Results**	**Ref**
Shin and Banerjee	2011	Li_2_CO_3_ (62%) + K_2_CO_3_ (38%)	SiO_2_ (10 nm), 1% wt.	Salt + water (1:100)	C_p_: +19% to +24%	[10]
				Nanoparticle dispersion in ultrasonic bath (100 min). Drying in hot plate (200°C)		
Shin and Banerjee	2011	BaCl_2_ (34%) + NaCl (13%) + CaCl_2_ (40%) + LiCl (13%)	SiO_2_, 1% wt.	Salt + water (1:100)	C_p_: +14.5%	[16]
				Nanoparticle dispersion in ultrasonic bath (100 min). Drying in hot plate (200°C)		
Lu and Huang	2013	NaNO_3_ (60%) + KNO_3_ (40%)	Al_2_O_3_ (13 nm, 90 nm), 0.9% to 4.6% wt.	Salt + water (1:100)	C_p_: -10%, 4.6% wt.	[17]
				Nanoparticle dispersion in ultrasonic bath (100 min). Drying in hot plate (105°C)		
Tiznobaik and Shin	2013	Li_2_CO_3_ (62%) + K_2_CO_3_ (38%)	SiO_2_ (5 to 60 nm), 1% wt.	Salt + water (1:100)	C_p_: +23% to +29%	[11]
				Nanoparticle dispersion in ultrasonic bath (100 min). Drying in hot plate (200°C)		
Tiznobaik and Shin	2013	Li_2_CO_3_ (62%) + K_2_CO_3_ (38%)	SiO_2_ (10 nm), 1% wt.	Salt + water (1:100)	C_p_: +26%	[12]
				Nanoparticle dispersion in ultrasonic bath (100 min). Drying in hot plate (200°C)	C_p_: +3% (addition NaOH)	
Shin and Banarjee	2013	Li_2_CO_3_ (62%) + K_2_CO_3_ (38%)	SiO_2_ (2 to 20 nm), 1% wt.	Salt + water (1:100)	Segregation	[13]
				Nanoparticle dispersion in ultrasonic bath (100 min). Drying in hot plate (100°C)	C_p_: +124% (zone A)	
					C_p_: +0% (zone B)	
Dudda and Shin	2013	NaNO_3_ (60%) + KNO_3_ (40%)	SiO_2_ (5 nm, 10 nm, 30 nm, 60 nm), 1% wt.	Salt + water (1:100)	C_p_: +10% (5 nm) +13% (10 nm), +21% (30 nm), +28% (60 nm)	[18]
				Nanoparticle dispersion in ultrasonic bath (100 min). Drying in hot plate (200°C)		
Chieruzzi et al.	2013	NaNO_3_ (60%) + KNO_3_ (40%)	SiO_2_ (7 nm), Al_2_O_3_ (13 nm), TiO_2_ (20 nm), SiO_2_ + Al_2_O_3_(2 to 200 nm), 0.5% to 1.5% wt.	Salt + water (1:100)	C_p_: +22.5% (SiO_2_ + Al_2_O_3_ (2 to 200 nm 1.0% wt.))	[19]
				Nanoparticle dispersion in ultrasonic bath (100 min). Drying in hot plate (200°C)	H: +15% (all np except TiO_2_, 1% wt.)	
Jo and Banarjee	2014	Li_2_CO_3_ (62%) + K_2_CO_3_ (38%)	Graphite (50 nm) + gum arabic	Salt + water (1:100)	Segregation	[14]
				Nanoparticle dispersion in ultrasonic bath (120 min). Drying in hot plate (200°C)	C_p_: +100% (material 1)	
					C_p_: +33% (material 2)	
Shin and Banarjee	2014	Li_2_CO_3_ (62%) + K_2_CO_3_ (38%)	Al_2_O_3_ (10 nm), 1% wt.	Salt + water (1:100)	C_p_:+32%	[15]
				Nanoparticle dispersion in ultrasonic bath (120 min). Drying in hot plate (100°C)		
Ho and Pan	2014	NaNO_3_ (7%) + KNO_3_ (53%) + NaNO_2_ (40%)	Al_2_O_3_ (<50 nm), 0.016% to 1% wt.	Salt + nanoparticle aqueous suspension (20% wt.)	C_p_: +19.9% (0.063% wt.)	[20]
				Nanoparticle dispersion by mechanic stirring at high temperature (180 min.)		
Present work		NaNO_3_ (60%) + KNO_3_ (40%)	SiO_2_ (12 nm), 0.5% to 2.0% wt.	Salt + water (1:10)	C_p_: +25% (1.0% wt.)	-
				Nanoparticle dispersion in ultrasonic probe (5 min). Drying in hot plate (100°C)		

Two synthesis methods have been proposed. The first one was proposed by Shin and Banerjee in 2011 [9]. In this method, salts are first dissolved in water at a low concentration (1:100 ratio) and then nanoparticles are dispersed by means of ultrasounds. Finally, the suspension is dried in a hot plate. Most of the published works use this method [9-19]. An alternative synthesis method has recently been proposed by Ho and Pan [20]. In this method, nanoparticles are produced directly by chemical reaction in an aqueous suspension at high concentration and the suspension is added directly to the salt before melting. Nanoparticle dispersion is obtained by mechanical stirring when the salt is melted at a high temperature. In both synthesis methods, an optimum nanoparticle concentration is found (1% wt. for Shin and Banerjee method [19] and 0.063% wt. for Ho and Pan method [20]). Experimental data shows that nanoparticle dispersion plays a major role in the Cp increment since it is related with the interaction of the salt components with the nanoparticle surface. It has to be mentioned that this behavior can only be found in salt-based nanofluids and not in water- and ethylene glycol-based nanofluids.

The mechanism underlying the enhancement of Cp in salt-based nanofluids is still a matter of continuous research. At present, a theoretical model based on semisolid layering of the salt ions around nanoparticle surface has been developed in order to explain the increment in Cp in salt-based nanofluids. The nanoparticles introduced into the salt act as nucleation points for the crystallization of a new phase around them, having higher thermophysical properties than the bulk molten salts. Salt ions are ordered around nanoparticles due to electrostatical interactions, forming fractal-like structures. The semisolid layering approach is in accordance with the increment observed in Cp and the available nanoparticle surface area; however, more experimental work is needed in order to check and refine the model [21-23].

In this work, salt-based nanofluid based on solar salt and SiO_2_ nanoparticles prepared by the Shin and Banerjee method was investigated. The optimal nanoparticle concentration was found and the stability of the nanofluid with thermal cycles and high-temperature conditions were tested. Finally, the dependence of the specific heat capacity of the nanofluid with the available surface of nanoparticles was demonstrated.

## Methods

### Nanofluid synthesis procedure

Solar salt (60% NaNO_3_ + 40% KNO_3_) has been used as a base material since it is the actual material used in sensible heat TES systems in CSP plants. The salts were purchased from Sigma-Aldrich (St. Louis, MO, USA). SiO_2_ nanoparticles in powder form with 12-nm primary average diameter from Sigma-Aldrich (St. Louis, MO, USA) were selected.

Solar salt-based nanofluids with nanoparticle mass concentrations of 0.5%, 1%, 1.5%, and 2% were prepared. The nanofluid synthesis procedure was based on the Shin and Banerjee method, covering four steps: binary salt was prepared by mixing 60 parts of NaNO_3_ with 40 parts of KNO_3_ in solid state, the binary salt was diluted in distilled water (1:10 ratio), the nanoparticles were added and dispersed using a ultrasound probe (Sonopuls HD2200, Bandelin, Italy) during 5 min, and, finally, the suspension was dried in a hot plate at 100°C. Typical drying time of the sample is 60 min. The maximum drying temperature is 100°C in order to avoid boiling and, thus, nanoparticle agglomeration [15]. Air forced convection is used during the drying process in order to reduce evaporation time and improve sample homogeneity. Air convective stream is applied to the upper part of the sample. All components were measured in an analytical balance with ±0.1-mg precision (Mettler Toledo, type AB104-S, Greifensee, Switzerland).

### Differential scanning calorimetry

Differential scanning calorimetry (DSC) tests were performed on a Mettler Toledo DSC 822E/400 (Mettler Toledo, Greifensee, Switzerland) in order to obtain the specific heat capacity of the solar salt and solar salt-based nanofluids. The specific heat capacity was determined using a standard protocol established by American Standard Test Method (ASTM E1269) [24]. According to this protocol, the difference in heat flow between an empty pan (standard aluminum pan) and a reference material (sapphire disk) was recorded as a function of sample temperature from the DSC. The measurements were repeated for the same thermal cycle for the samples (solar salt and nanofluids). The specific heat capacity is calculated from the equation:

(2) $${Cp}_{s}={Cp}_{st}\frac{\Delta q_{s}m_{st}}{\Delta q_{st}m_{s}}$$

where Cp is the specific heat capacity, *Δq* is the heat flow difference between the sample and the empty pan, and *m* is the mass. Subscript *s* indicates samples and st indicates the standard material (sapphire). The heat flow differences were obtained by subtracting the baseline heat flow (empty pan) from the heat flow of the sapphire and the sample. Uncertainty analysis of Equation 2 provides a maximum uncertainty of ±3.5%. Uncertainty values were obtained from equipment technical specifications (mass and heat flows) and from curve fitting of the sapphire specific heat capacity.

The measurement protocol was as follow: the dried sample was introduced in an empty pan and dried at 150°C during 30 min in order to eliminate moisture. Then, the sample was kept at 300°C during 5 min in order to melt it inside the pan (melting temperature of solar salt is 222°C). Finally, the sample was introduced in the DSC and the following thermal cycle was used: isothermal of 10 min at 160°C, heating from 160°C to 420°C at 20°C/min, isothermal of 5 min at 420°C, cooling from 420°C to 160°C at 40°C/min. Only the heating ramp was used for the specific heat measurements. The heat flow data of the first thermal cycle (sample, empty pan, and sapphire) was discarded because it can be shifted due to thermal non-equilibrium issues. The measurement reproducibility was checked by repeating the calorimetric measurements five times in selected samples. The heat flow reproducibility agrees with the measurement uncertainty (±3.5%).

### Scanning electron microscopy

The dispersion of nanoparticles in solid samples was evaluated using a field emission scanning electron microscope (SEM) (JEOL, 7001F, Akishima-shi, Japan). Specimens of selected samples measured in the DSC were metallized in a thermal evaporator. Secondary electron images and digital image processing were used to characterize the nanoparticle clusters in the solid salt and measure the available nanoparticle surface area.

### Dynamic light scattering

Furthermore, nanoparticle dispersion was characterized in aqueous suspension using a dynamic light scattering (DLS) system (ZetaSizer nano ZS, Malvern Instruments, Ltd. Malvern, UK). Specimens of selected samples measured in the DSC were dissolved in ultrapure water (1-mg salt/1-ml water) and measured by DLS. The Brownian motion of particles in suspension causes laser light to be scattered at different intensities, depending on the particle size. Analysis of these intensity fluctuations provides the velocity of the Brownian motion and hence the particle size using the Stokes-Einstein relationship. Tests were performed with a 173° scattering angle.

### Thermogravimetric analysis

The solar salt and solar salt-nanofluid stability at high temperature was measured by thermogravimetric analysis (TGA) system (Mettler Toledo, TGA1, Greifensee, Switzerland). The sample decomposition is characterized by the mass loss measured with a high-precision balance in nitrogen atmosphere and controlled sample temperature conditions from 250°C to 750°C with a heating ramp of 20 K min^-1^.

## Results and discussion

### Calorimetric analysis

**Figure 1:**
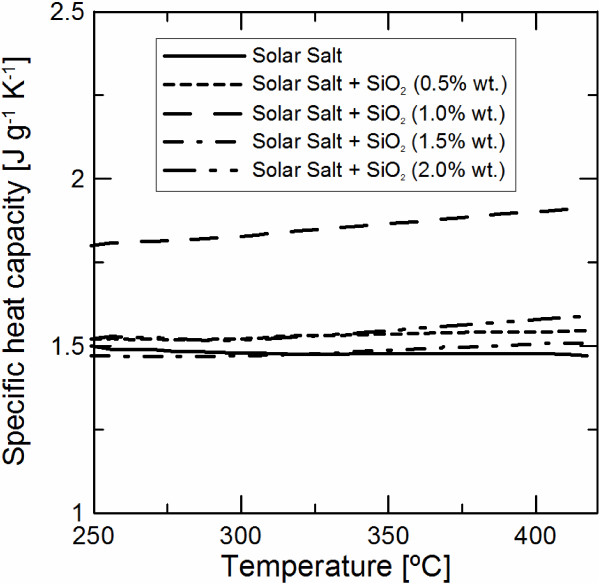
Average specific heat capacity dependence with temperature.

Specific heat measurements have been performed by the DSC from 160°C to 420°C. Solar salt and solar salt-based nanofluids with SiO_2_ nanoparticles and concentrations of 0.5, 1.0, 1.5 and 2.0 wt. %. (five samples each) have been characterized. Figure 1 shows the average specific heat dependence with temperature of the solar salt and salt-based nanofluids in liquid state. Only results from 250°C to 420°C have been considered since solar salt is used in TES system as a sensible heat material in liquid phase.

It is possible to observe that the results obtained for solar salt are similar to those reported from different researchers with an average value of 1.45 to 1.55 J g^-1^ K^-1^ and a weak dependence with temperature [25].

Table 2 shows the specific heat capacity results for every individual sample averaged in Figure 1. Average values from 250°C to 420°C have been considered. The maximum enhancement of the specific heat capacity was obtained for 1% wt. salt-based nanofluid, as reported in previous works using the Shin and Banerjee preparation method (see Table 1). In addition, the obtained enhancement, almost 25%, is similar to those reported in previous works. Salt-based nanofluids with other nanoparticle concentrations present weak specific heat capacity increment, with enhancement values near the accuracy given by the DSC system. In all the cases, the specific heat capacities of the nanofluid present higher values than those predicted by the mixture rule (Equation 1).

**Table 2:** Average specific heat capacity for a temperature range of 250°C to 420°C

**Specific heat capacity (J g**^**-1 **^**K**^**-1**^**)**					
**Sample (number)**	**Solar salt**	**Solar salt +0.5% SiO**_**2**_	**Solar salt +1.0% SiO**_**2**_	**Solar salt +1.5% SiO**_**2**_	**Solar salt +2.0% SiO**_**2**_
1	1.40	1.53	1.85	1.61	1.64
2	1.57	1.55	1.95	1.54	1.65
3	1.39	1.52	1.85	1.41	1.34
4	1.46	1.62	1.78	1.38	1.54
5	1.58	1.43	1.82	1.48	1.49
Average value	1.48	1.53	1.85	1.51	1.53
Standard deviation	0.09	0.07	0.06	0.09	0.12
Enhancement [%]	-	+3.41	+25.03	+2.00	+3.69

### Thermal cycling and high-temperature stability tests

**Figure 2:**
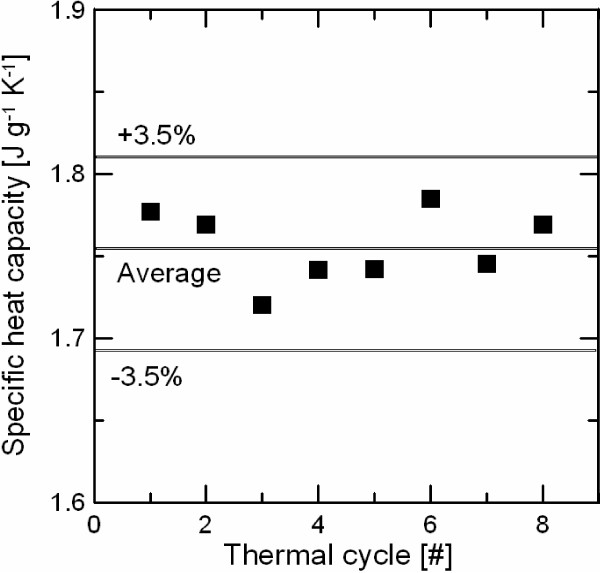
Specific heat capacity of 1% wt. salt-based nanofluid dependence with thermal cycling.

**Figure 3:**
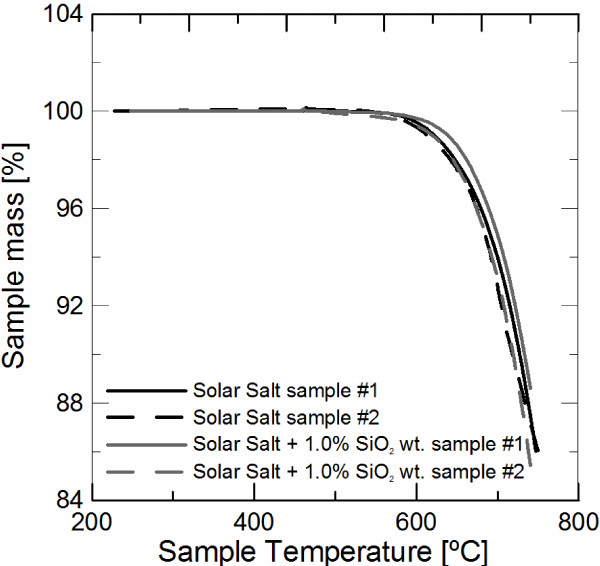
TGA measurements of solar salt and 1% wt. salt-based nanofluid.

Solar salt-based nanofluid (1% wt.) stability with thermal cycling has been measured by DSC by repeating eight heating and cooling cycles between 160°C and 420°C over the same sample. Figure 2 shows the specific heat measurements for a temperature range of 250°C to 420°C performed during the heating ramps. It is possible to observe that the measurements are scattered around an average value of 1.751 J g^-1^ K^-1^ with no tendency. Standard deviation of the measurements is below the accuracy given by the DSC system, showing the stability of the salt-based nanofluid with thermal cycling.High-temperature stability tests have been performed by TGA measurements. Figure 3 shows the sample mass loss of two samples of solar salt and 1% wt. salt-based nanofluid measured by TGA. As can be observed in the figure, solar salt and nanofluids present a similar decomposition curve. All measured samples have a maximum mass loss below 0.3% for 560°C (maximum working temperature of solar salt) and below 8% for 700°C, showing the stability of the salt-based nanofluid at high-temperature conditions.

### Nanoparticle dispersion

**Figure 4:**
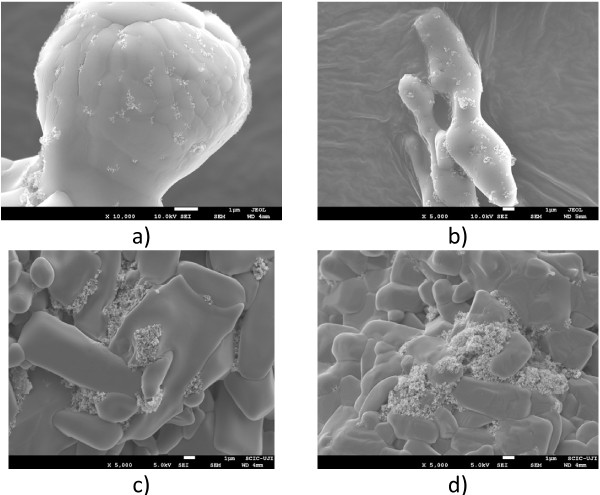
Example of SEM images: (a) 0.5% wt., (b) 1.0% wt., (c) 1.5% wt., and (d) 2.0% wt.

**Figure 5:**
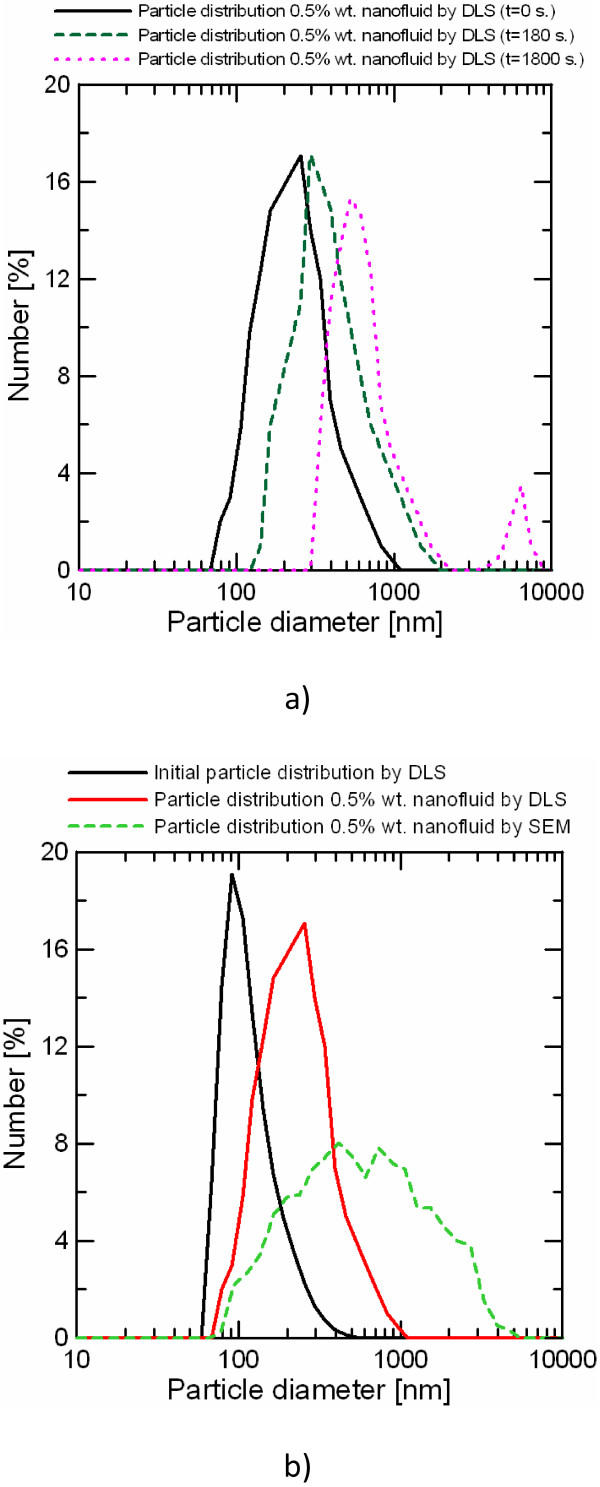
**Time evolution and comparison of particle size distributions. (a)** Time evolution of particle size distribution obtained by DLS. **(b)** Comparison of particle size distributions obtained by DLS and SEM images.

Several works have proposed the nanoparticle dispersion or available particle (nanoparticle/nanoparticle cluster) surface area, S_p_, as a key parameter for the salt-based nanofluid specific heat capacity enhancement. However, it is not possible to measure S_p_ in real conditions due to the high-temperature working conditions of inorganic salts. In this way, only approximated nanoparticle surface characterization in simplified experimental conditions are possible. In this work, two different evaluations of S_p_ are performed:

– The first evaluation is similar to that proposed by Ho and Pan [20] and it is based on imaging processing of SEM images. Several images of solid nanofluid samples are obtained and particles (nanoparticles and nanoparticle clusters) are identified and measured by digital imaging processing techniques. In the case of nanoparticle clusters, their shape is fitted to a sphere in order to perform the surface calculation. The maximum resolution (×10,000 images) is 10 nm/pixel. Consequently, isolated nanoparticles will be not detected (the minimum detectable nanoparticle cluster size is estimated in 30 nm by image processing tests). However, the minimum nanoparticle cluster (particle) size that can be expected using the Shin and Banerjee synthesis method is higher as it will be shown in the next paragraphs. The main drawbacks of this method are: nanoparticle dispersion can be different in solid and melted state, measurements are biased to high-size clusters since they are easier to be identified, the amount of characterized sample is very small, and it is a time-consuming process since particle identification is made one by one. Thus, particle dispersion measured by SEM images can only be considered as a first approximation of the real particle distribution found in the melted salt. In this work, at least 500 particles where identified and measured for every salt-based nanofluid composition. Figure 4 shows examples of SEM images for every salt-based nanofluid tested.

– The second evaluation is based on the DLS technique. Direct light scattering measurements in melted salt-based nanofluids are not possible due to temperature experimental limitations. In order to perform the measurements, the samples characterized by DSC were dissolved in ultrapure water, and the suspension was measured by DLS. In this case, also the shape of the cluster was fitted to a sphere. The data rate with this method depends on the nanoparticle concentration and it was established between 150 and 350 ksamples/s. The main drawback of this method is that nanoparticle dispersion can be different in the suspension than in the melted state. Consequently, it can only be considered as a first approximation of the dispersion found in the melted salt at high-temperature conditions. However, the measurements are fast and the amount of characterized sample and measured particles is much higher than with the first method. Figure 5a shows the time evolution of particle distribution of 0.5% wt. salt-based nanofluid measured by this method. The curve labeled as *t* =0 s corresponds to the distribution measured right after the salt-based nanofluid dissolution in water. It is possible to observe that the distribution ranges from 70 nm to 1,000 nm with a peak value near 240 nm. In the next two distribution curves, particle agglomeration can be observed. After 180 s, the distribution ranges from 100 nm to 1,100 nm with a peak value of 300 nm. Finally, after 1,800 s, a bimodal distribution is measured with peak values of 500 nm and 6,500 nm.

Figure 5b shows the particle size distributions of the 0.5% wt. salt-based nanofluids measured by both SEM and DLS methods as well as the distribution obtained by DLS of a suspension of ultrapure water and a low concentration of nanoparticles dispersed by the ultrasound probe (initial particle distribution). It is possible to observe that the initial particle distribution average value is 91.3 nm, with no clusters smaller than 60 nm. Ultrasound probe could not break completely the nanoparticle clusters found in powder form to the primary diameter of 12 nm even if sonication time was extended. It is expected that the average value of the initial particle distribution obtained with ultrasound bath will be higher since its ultrasonic power is much lower than the probe. The particle size distribution of the nanofluid sample measured by DLS corresponds to the one labeled as *t* =0 s. in Figure 5a. This curve has been selected since it corresponds to the distribution with lowest peak value in Figure 5a, but with higher average and standard deviation values than those corresponding to the initial particle distribution. It is considered that the distributions measured later are affected by particle agglomeration in water. This distribution shows an average value similar to that in the initial particle distribution, but a considerable higher width and with clusters up to 1,100 nm. This change in the particle size distribution could be produced by nanoparticle aggregation during the nanofluid drying and solidification process and time in liquid state (the sample has been measured previously by DSC), but not during the dissolution of the sample in water and first measurements, since these processes take few seconds. The particle size distribution of the nanofluid sample measured by SEM shows a considerable higher average value and width. Differences between the particle size distributions measured by both methods could be due to big cluster bias in SEM images and partial cluster disintegration during sample preparation for the DLS measurement.

**Figure 6:**
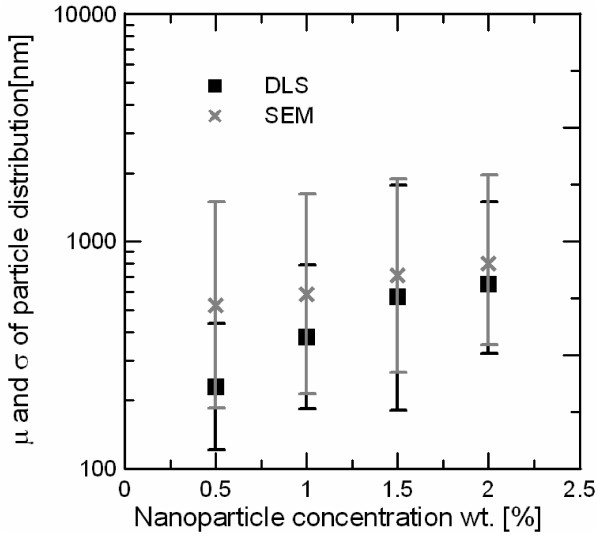
Particle average size and standard deviation of salt-based nanofluids measured by DLS and SEM.

The particle size distribution of the four salt-based nanofluid compositions has been evaluated by both methods. For comparison purposes, all the distributions have been fitted to a modified normal distribution given by,

(3) $$PDF=\frac{1}{\sqrt{2\pi }\sigma }e^{-\frac{{\left(Ln\left(x\right)-\mu \right)}^{2}}{2\sigma^{2}}}$$

where $1/\sqrt{2\pi }$, μ, σ, and *x* are an integration constant, the mean, standard deviation, and x-axis coordinate in nanometer of the modified normal distribution function (PDF), respectively. Figure 6 shows the mean values and standard deviations for the four nanoparticle concentrations. It is possible to observe that in both methods, the average particle size increases with nanoparticle concentration as reported in water-based nanofluids [5]. In addition, DLS mean and standard deviation values are always lower than those obtained by SEM. However, due to the high values of the standard deviations obtained by SEM, the distributions obtained by both methods overlap. It can be considered that the particle distributions obtained by both methods are only a first approximation of the real particle distribution found in the salt-based nanofluid in the melted state, but they can be used for demonstration purposes. The nanoparticles are always found agglomerated in clusters and the cluster size depends on the nanoparticle concentration. This dependence can be found in water-based nanofluids [6]. Large clusters can lead to nanofluid precipitation, limiting its practical applicability. However, a DLS system working at very high-temperature conditions is needed in order to confirm this fact.

The available particle surface area, S_p_, is calculated from the particle size distribution by,

(4) $$S_{p}=\theta \sum_{i=1}^{N}N_{i}A_{i}$$

where *θ* is the nanoparticle mass fraction, *N* is the total number of bins of the distribution, *N*_*i*_ the probability of the i-bin, and *A*_*i*_ is the surface area of a 1 g of nanoparticles with a diameter that corresponds to the i-bin. The units of the particle surface area calculated by Equation 4 are square meter per gram of salt.

**Figure 7:**
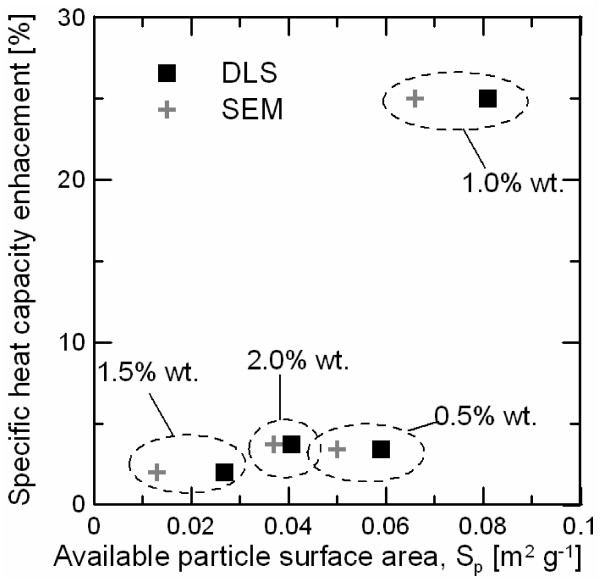
Specific heat capacity enhancement dependence with available particle surface area.

Figure 7 shows the dependence of the increment in the specific heat capacity with the available particle surface area measured by both methods. It is possible to observe that the specific heat enhancement is highly dependent on the available particle surface area. Low values of S_p_ correspond to either low solid content with few particles or high solid content with agglomeration of particles. In these cases, the interaction between salt ions and nanoparticle surface is very low to promote enhancement of specific heat. However, it can be established a minimum S_p_ at which interactions become important, thus providing an enhancement of the thermal capacity for higher values. These results prove that the mechanism involved in the process is based on a surface phenomenon and there is an optimal concentration of nanoparticles corresponding to a S_p_ in the range of specific heat enhancement.

## Conclusions

In the present study, salt-based nanofluids based on eutectic mixtures of nitrate with SiO_2_ nanoparticles were studied. Four weight fractions were evaluated (0.5, 1.0, 1.5, and 2 wt. %). The synthesized samples were studied by means of DSC, TGA, SEM, and DLS techniques.

Calorimetry analysis revealed an increase in Cp of the salt-based nanofluids compared to the base salt. Several samples were evaluated for each concentration to the reproducibility of the measurements. An optimum nanoparticle concentration was obtained at 1.0 wt. %, with an enhancement in the Cp of 25% for a temperature range from 250°C to 420°C.

The thermal cycling stability of the salt-based nanofluid was proved by several heating and cooling cycles of the samples. High-temperature stability of the nanofluids was confirmed by means of TGA measurements of up to 750°C.

The available nanoparticle surface area was evaluated by two different methodologies in the salt-based nanofluids: one processing SEM images of solid nanofluid samples and the other dissolving the already melted salt-based nanofluid and using DLS techniques. The measured Cp enhancements for the different concentrations were related with the calculated available nanoparticle surface areas, suggesting that this is a key parameter in the specific heat capacity increase.

The increase in the specific heat capacity of the salt-based nanofluid could significantly reduce the cost of the thermal energy storage media in CSP and therefore the cost of the generated electricity. The identification of a mechanism controlling this increase could guide and optimize this cost reduction.

## Abbreviations

DLS: dynamic light scattering; CSP: concentrated solar power; DCS: differential scanning calorimetry; SEM: scanning electron microscopy; TES: thermal energy storage; TGA: thermogravimetric analysis; C_P_: specific heat capacity; S_np_: nanoparticle surface area; S_p_: available particle surface area.

## Competing interests

The authors declare that they have no competing interests.

## Authors’ contributions

The manuscript was written through contributions of all authors. All authors read and approved the final manuscript.
